# Determination of calcium, iron, and selenium in human serum by isotope dilution analysis using nitrogen microwave inductively coupled atmospheric pressure plasma mass spectrometry (MICAP-MS)

**DOI:** 10.1007/s00216-024-05274-0

**Published:** 2024-04-08

**Authors:** Zengchao You, Alexander Winckelmann, Jochen Vogl, Sebastian Recknagel, Carlos Abad

**Affiliations:** 1 Bundesanstalt für Materialforschung und -Prüfung (BAM), Department 1 Analytical Chemistry; Reference Materials, Richard-Willstätter-Straße 11, 12489 Berlin, Germany; 2 Humboldt-Universität Zu Berlin, Department of Chemistry, Brook-Taylor-Straße 2, 12489 Berlin, Germany

**Keywords:** Nitrogen microwave inductively coupled atmospheric pressure mass spectrometry, Isotope dilution, Human serum, Calcium, Iron, Selenium

## Abstract

In this study, we demonstrate the applicability of nitrogen microwave inductively coupled atmospheric pressure mass spectrometry (MICAP-MS) for Ca, Fe, and Se quantification in human serum using isotope dilution (ID) analysis. The matrix tolerance of MICAP-MS in Na matrix was investigated, revealing that high Na levels can suppress the signal intensity. This suppression is likely due to the plasma loading and the space charge effect. Moreover, ^40^Ca and ^44^Ca isotopic fractionation was noted at elevated Na concentration. Nine certified serum samples were analyzed using both external calibration and ID analysis. Overestimation of Cr, Zn, As, and Se was found in the results of external calibration, which might result from C-induced polyatomic interference and signal enhancement, respectively. Further investigations performed with methanol showed a similar enhancement effect for Zn, As, and Se, potentially supporting this assumption. The mass concentrations determined with ID analysis show metrological compatibility with the reference values, indicating that MICAP-MS combined with ID analysis can be a promising method for precise Ca, Fe, and Se determination. Moreover, this combination reduces the influence of matrix effects, broadening the applicability of MICAP-MS for samples with complex matrixes.

**Graphical abstract:**

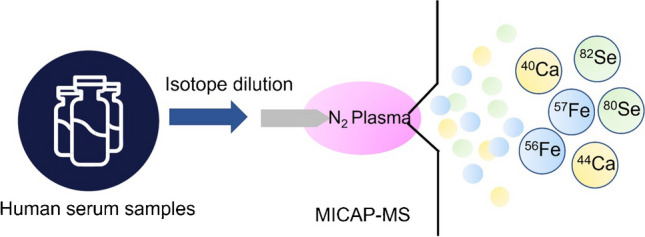

**Supplementary Information:**

The online version contains supplementary material available at 10.1007/s00216-024-05274-0.

## Introduction

As essential components of enzymes and hormones, trace elements play an indispensable role in various biological systems. For example, Ca is needed for bone mineralization, heart rate regulation, and nerve impulse regulation [1–3]. Fe participates in a wide variety of metabolisms, like oxygen transport and neurotransmitter myelin synthesis [4–6]. Se is vital for antioxidant selenoprotein synthesis, protecting the body against the oxidative stress [7–9]. Accurate characterization of trace elements in the human body enables not only the monitoring of various physiological mechanisms but also assists in various clinical practices such as nutritional assessment and disease diagnosis.

Inductively coupled plasma mass spectrometry (ICP-MS) stands out as a preeminent method for trace elemental analysis due to its exceptional sensitivity and multielement capability. However, direct analysis of serum samples by ICP-MS encounters matrix effects from Na [10–12], organic species [13–15], and polyatomic interferences. These effects have the potential to lead to signal suppression and enhancement, thereby introducing distortions in the analysis. The capability for isotope ratio measurements in ICP-MS facilitates the application of isotope dilution (ID) analysis [16]. By utilizing an isotopically enriched spike that shares the same matrix as the sample, it becomes feasible to eliminate matrix effects originating from the serum matrix. Furthermore, ID has the potential to mitigate errors related to sample preparation, thereby leading to increased precision and accuracy. Over the past few decades, ICP-MS combined with ID analysis has emerged as one of the most widely adopted techniques for trace elemental analysis in serum [17, 18].

However, Ar as plasma gas in ICP-MS hinders the ID analysis of certain elements by Ar-related interferences, particularly in the case of Ca, Fe, and Se. Their most abundant isotopes are interfered by ^40^Ar^+^ (^40^Ca), ^40^Ar^16^O^+^ (^56^Fe), and ^80^Ar_2_^+^ (^80^Se), respectively. Additionally, the intense ion beam generated by these interferences may cause ion/electron scattering in the detector, resulting in non-spectral interferences to isotopes with similar mass-to-charge ratios [19, 20]. Various approaches have been implemented to address this issue, for example, collision cell [17], dynamic reaction cell [21], and “cold” plasma conditions [22]. In addition to these methods, one approach that can fundamentally solve this problem is to replace the Ar plasma gas with N_2_.

In the 1990s, Hitachi et al*.* first introduced the coupling of N_2_-based microwave-induced plasma with mass spectrometry (MIP-MS), and this instrument was proved to be effective in various application areas [23, 24]. In 1998, MIP-MS was combined with ID to determine the Se concentration in serum by Furuta et al*.* [25]. They indicated that the results agreed well with the reference values, whereas the detection sensitivity of N_2_-MIP is about one order of magnitude lower than that of Ar-ICP. Three years later, Majidi et al*.* coupled MIP with time-of-flight mass spectrometry (TOF–MS) to measure the Ca isotopes and isotope ratios. They showed that all Ca isotopes can be measured with this method. However, the precision is poorer than with normal ICP-MS [26]. Compared to MIP-MS, microwave inductively coupled atmospheric-pressure plasma mass spectrometry (MICAP-MS) exhibited greater sensitivity and precision due to its higher plasma power. It has proven to be a promising alternative to ICP-MS in different fields [27–29]. However, its performance for ID analysis has not been reported so far.

In this work, we employed MICAP-MS to quantify the concentrations of Ca, Fe, and Se in human serum using external calibration and ID analysis, utilizing the isotope ratios ^40^Ca/^44^Ca, ^57^Fe/^56^Fe, and ^82^Se/^80^Se. Nine reference serum samples were digested and analyzed. The results obtained from both methods were compared and validated against certified values. Since serum has a remarkable level of Na, the performance of MICAP-MS under varying Na matrix concentrations was studied. Furthermore, the effect of organic species was also investigated and discussed.

## Experimental

### Materials and samples

The reference serum samples analyzed included NIST 909C (National Institute of Standards and Technology, USA), BCR-304, BCR-637, BCR-638, BCR-639 (Joint Research Centre, Belgium), Seronorm L-1, Seronorm L-2 (Sero As, Norway), ClinChek Level 1, ClinChek Level 2 (Recipe Chemicals + Instruments GmbH, Germany). The certified mass concentrations of the elements contained in the samples are listed in Table S1. High-purity deionized water with a resistivity of 18 MΩ cm obtained from a Milli-Q system (Merck Millipore, Germany) was used throughout the experiments. HNO_3_ (Merck, Germany) was used after purification by subboiling distillation in PFA containers.

For ID analysis, the spike solution used for Se determination was prepared by diluting the liquid isotope standard VHG-LIS82Se-50 (VHG Labs, USA) by a factor of 100. After dilution, this spike solution has a Se mass concentration of around 100 µg L^−1^ (99.72% ^82^Se). The spike solution utilized for Fe determination was prepared using the liquid isotope standard IRMM-620 (IRMM, Belgium), which contains approximately 11 mg L^−1^ of Fe with a ^57^Fe isotope abundance of 95.19%. In the case of calcium determination, the spike solution was prepared from the solid ^44^Ca enriched isotope material ISOFLEX-Ca-44 (ISOFLEX, USA), which is in the form of carbonate and has a ^44^Ca isotope abundance of 99.2%. To prepare the spike solution, 17.63 mg standard was weighted and dissolved with 2% HNO_3_. After dissolution, the Ca spike solution has a ^44^Ca mass concentration of 529 mg L^−1^. To ensure that the ratios of ^40^Ca/^44^Ca and ^57^Fe/^56^Fe after spiking were close to 1, while the ratio of ^82^Se/^80^Se is about to 1.5, the masses of the spike solutions to be added were calculated according to the natural abundance and the mass concentration of the corresponding elements in the samples. The mass concentrations and weighted masses of the spike solutions are shown in Table S2. For the reverse ID analysis, spike solutions were mixed with single-element solutions diluted from the ICP stock solutions (Merck AG, Germany), respectively (see Table S3). A solution containing 100 mg L^−1^ Ca, 10 mg L^−1^ Fe, and 100 µg L^−1^ Se was used for sample bracketing. To avoid the memory effect of Se, two rinsing steps with a rinse time of 60 s were applied subsequently between each sample.

External calibrations were carried out using multielement solutions prepared from single-element ICP stock solutions. To mimic the matrix in the serum samples, 50 mg L^−1^ NaCl (Merck AG, Germany) was added to each standard. Six calibration levels were applied for all the elements, and the concentration of the standards ranged from 0.1 to 500 µg L^−1^. ^6^Li, ^45^Sc, ^89^Y, ^115^In, ^159^ Tb, and ^209^Bi were used as internal standards (IS) in each calibration standard and sample (see Table S4). To investigate the matrix effect of Na and C, matrix-matched solutions with increasing concentrations were prepared with NaCl and Methanol (Merck AG, Germany), respectively.

### Serum sample preparation

0.6 g of each serum sample was mixed with a corresponding volume of spike solution and subjected to digestion with 4 mL 65% HNO_3_ and 2 mL 15% H_2_O_2_ (Merck AG, Germany). The reaction mixture was heated at 100 °C for 60 min in a microwave digester (Anton Paar Multiwave 5000, Germany). After cooling, it was diluted to 25 mL with 2% HNO_3_ and used as stock solution. For the determination of Ca, the stock solution was diluted by a factor of 10. Undiluted stock solution was applied for Fe and Se determination due to the low Se mass concentration in the serum samples.

### Instruments

A PlasmaQuant MS Elite quadrupole mass spectrometer (Analytik Jena GmbH, Germany) modified with a MICAP plasma source (Radom Research & Development, USA) was used for all measurements. Nitrogen (N_2_ purity ≥ 99.999%, Linde AG, Germany) was used as general nebulizer, auxiliary, and plasma gas. Samples were transported to a concentric nebulizer (MicroMist, USA) using a peristaltic pump at a liquid uptake rate of approximately 400 µL min^−1^. Larger aerosol particles were cut off by a cooled double-pass spray chamber. Aspect MS software (Analytik Jena GmbH, Germany) was used for data acquisition, including mass calibration, data processing, and plots. All torch parameters were optimized for high sensitivity and matrix tolerance. The optimized operation conditions are listed in Table 1.

**Table 1:** Optimized operation parameters used in MICAP-MS

MICAP-MS	
Plasma Power	1500 W
Nebulizer gas flow	1.1 L min^−1^ N_2_
Auxiliary gas flow	0.8 L min^−1^ N_2_
Plasma gas flow	9 L min^−1^ N_2_
Sampling depth	6 mm
Sampling cone	Pt 1.1 mm
Skimmer cone	Ni 0.5 mm

### Data analysis

For ID analysis, the mass concentrations of Ca, Fe, and Se in the serum samples were calculated according to Eq. (1) [16, 18], where *C* is the mass concentration, *M* is the molar mass, *m* is the mass, *x* is the amount fraction of the corresponding element in the sample, and *ρ* is the density determined through weighing (see Table S5).

1 $${C}_{sample}={C}_{spike}\frac{{\rho }_{sample}}{{\rho }_{spike}}\bullet \frac{{M}_{sample}}{{M}_{spike}\bullet {x}_{sample}}\bullet \frac{{m}_{spike}}{{m}_{sample}}\bullet \frac{{R}_{spike}-{R}_{mix}}{{R}_{mix}-{R}_{sample}}$$

*R* represents the isotope ratio, which is given by Eq. (2). *N* is the number of detected isotope atoms, which can be obtained by subtracting the background intensity from the signal intensity. *a* represents the less abundant isotope, while *b* denotes the more abundant isotope.

2 $$R=\frac{{N}_{a}}{{N}_{b}}$$

The mass concentration of the spike solution was determined using reverse ID by rearranging the equation for *C*_spike_ and setting *C*_sample_ as the certified concentration of the ICP standard solution. Expanded measurement uncertainty was estimated by multiplication of the standard deviation with factor two, for a confidence interval of 95%.

## Results and discussion

### Matrix tolerance of MICAP-MS at different Na concentrations

**Fig. 1:**
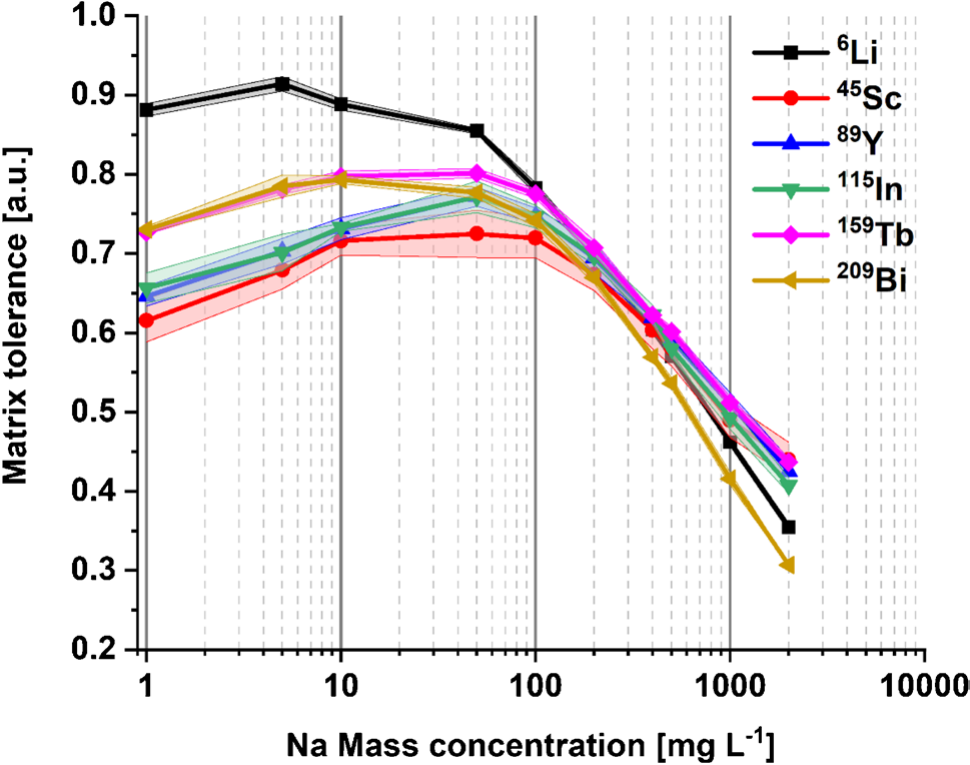
Matrix tolerance of ^6^Li, ^45^Sc, ^89^Y, ^115^In, ^159^ Tb, and ^209^Bi in 2% HNO_3_ with different NaCl mass concentrations. The lines with light color represent the standard deviation

Matrix tolerance serves as a metric for assessing an instrument’s robustness to matrix effects, which is described by the ratio of the signal intensity of an element/isotope obtained in the matrix containing solution to that from the element solution without matrix. The matrix tolerance of MICAP-MS at varying Na mass concentrations was investigated by measuring the intensity recovery of ^6^Li, ^45^Sc, ^89^Y, ^115^In, ^159^ Tb, and ^209^Bi in 2% HNO_3_ with NaCl mass concentrations ranging from 1 to 2000 mg L^−1^. The obtained results are shown in Fig. 1.

Na was noted to suppress the signal of all the elements measured. This is consistent with the reported results obtained with ICP-MS [10, 15], which indicates that easily ionizable elements (EIEs) like Na can reduce the plasma energy necessary for the ionization and may also lead to space charge effect within the interface region. Consequently, this culminates in a decrease in the number of detected analyte ions. The degree of the suppression is highly dependent on the Na mass concentration, which is about 15% at 50 mg L^−1^ for Li and between 25 and 30% for other elements. Li seems to be affected to a weaker extent, which might be due to its lower first ionization energy and smaller ionic mass. As Na mass concentration exceeds 100 mg L^−1^, the suppressing effect intensifies proportionally with the increase in Na mass concentration. As much as 60% suppression of the signal intensity was found with a Na mass concentration of 2 g L^−1^.

**Fig. 2:**
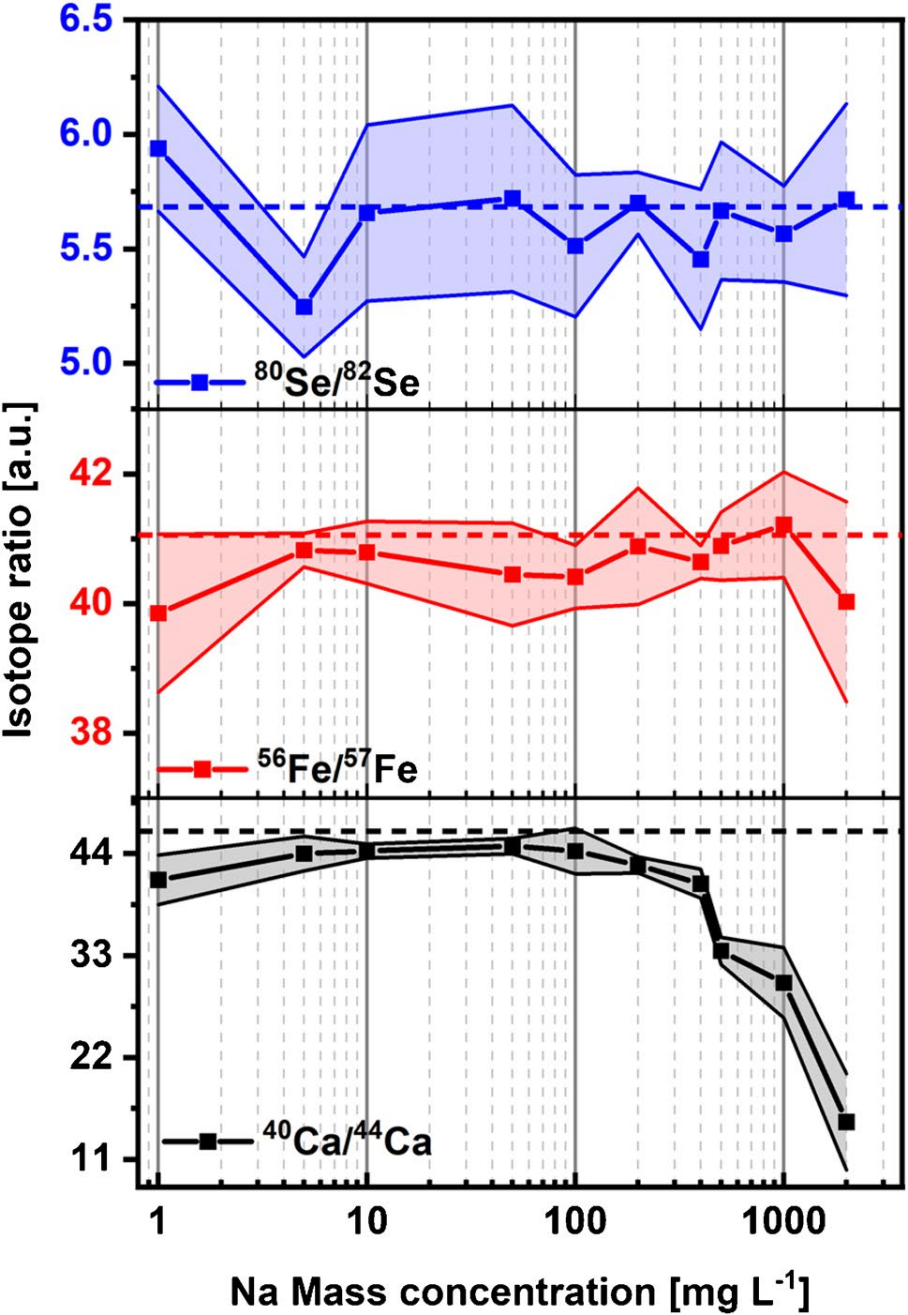
Variations in isotope ratios of ^40^Ca/^44^Ca, ^57^Fe/^56^Fe, and ^82^Se/^80^Se at different NaCl mass concentrations. The lines with light color represent the standard deviation, and the dashed lines indicate their natural isotope ratios

To investigate the influence of Na matrix on ID analysis, isotope ratios of ^40^Ca/^44^Ca, ^57^Fe/^56^Fe, and ^82^Se/^80^Se in solutions containing 100 µg L^−1^ Ca, Fe, Se, and increasing mass concentration of NaCl were determined. Figure 2 shows that isotope ratios of ^57^Fe/^56^Fe and ^82^Se/^80^Se were not significantly affected by the increasing Na mass concentration, which was almost consistent with the natural isotope ratios. Surprisingly, ^40^Ca/^44^Ca decreased significantly when the Na mass concentration exceeded 500 mg L^−1^. A possible explanation is that excessive Na ions enhanced the space charge effect in the skimmer cone regions, which improved the transmission of the heavier isotope and resulted in instrumental isotopic fractionation [30–32]. As a result, the isotope with lower ionic mass, in this case ^40^Ca, was less detected. Since the Na mass concentration in the serum samples was approximately 50 mg L^−1^, ID analysis of the serum samples with MICAP-MS should not be distorted by this issue.

### Characterization of the serum samples with external calibration

Besides ID analysis, nine reference serum samples were characterized with external calibration to validate the observed matrix tolerance of MICAP-MS in Na matrix. The higher-order, traceability, and commutability of the studied certified reference materials assure their representability as clinical human serum samples [33–36]. The choice of isotopes was based on their abundance and the absence of polyatomic interferences. Calibration standards were prepared with 2% HNO_3_ containing 50 mg L^−1^ NaCl to match the Na matrix in the serum samples. ^6^Li, ^45^Sc, ^89^Y, ^115^In, ^159^ Tb, and ^209^Bi were used as IS. The results obtained are the average values of the triplicate measurements.

**Fig. 3:**
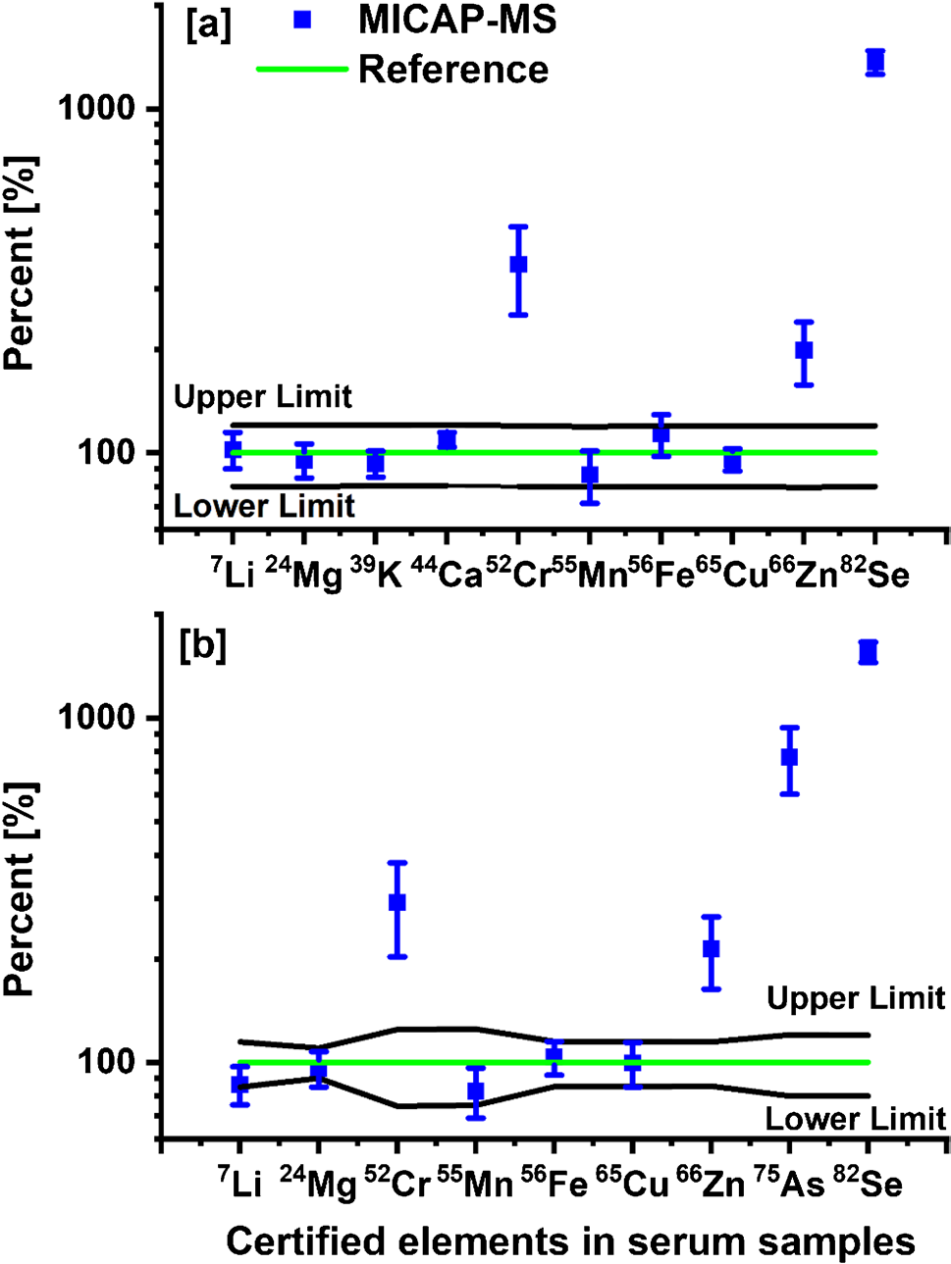
Percentage deviation in mass concentrations of the selected elements in serum samples: **a** Seronorm L-1 and **b** ClinChek-1 determined by external calibration (blue) compared to their reference values (green). The black lines represent the upper and lower limit of the reference ranges

**Fig. 4:**
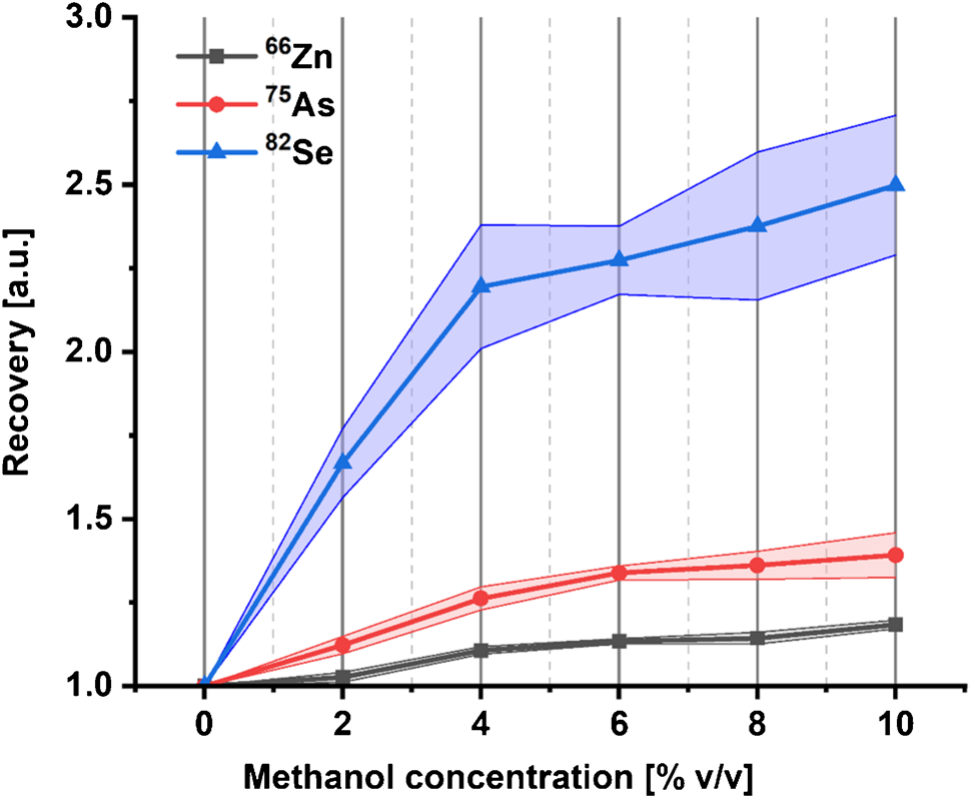
Matrix tolerance of ^82^Se, ^75^As, and ^66^Zn in 2% HNO_3_ with different methanol concentrations. The lines with light color represent the standard deviation

Figure 3 shows the results obtained with the serum samples Seronorm L-1 and ClinChek-1, demonstrating the percentage deviations in mass concentrations of the selected elements determined by external calibration compared to their reference values. For most of the elements, the results lie mostly within the reference ranges. However, the mass concentrations of Cr, Se, As, and Zn were significantly higher than their reference values. Similar results were also obtained with other serum samples (see Table S1). Although ^52^Cr suffers from polyatomic interference by ^40^Ar^12^C^+^, the effect of this interference should be negligible in MICAP-MS due to the low abundance of Ar in the N_2_ plasma gas. Other interferences might be ^35^Cl^16^O^1^H^+^ and ^40^Ca^12^C^+^. The isotope ratio of ^52^Cr/^50^Cr was observed 53% (29.6) higher than the natural isotope ratio (19.3). This might indicate that the main interference was ^40^Ca^12^C^+^ since ^50^Cr was interfered by ^35^Cl^15^N but not by Ca and C. The overestimation of Se, As, and Zn could be due to a signal enhancement effect since the same enhancement magnitude was noted with ^80^Se and ^78^Se. It was observed that the average C intensity in the serum samples was approximately 13 times higher than that in the blank, potentially indicating the presence of organic residue. Ionization of hard-to-ionize elements like Se, As, and Zn can significantly be improved by C ions in the conventional Ar plasma, because the electrons of these elements can be transferred to the C ions through a charge transfer reaction [13, 14, 37]. The Limits of detection (LOD) and limits of quantification (LOQ) of the applied method in Na matrix are shown in Table S6. To investigate whether C can enhance the intensity of these elements in MICAP-MS, matrix tolerance of ^82^Se, ^75^As, and ^66^Zn in 2% HNO_3_ with methanol concentrations ranging from 0 to 10% was measured using MICAP-Ms and the obtained results are shown in Fig. 4.

It can be observed that the signal intensities of Se, As, and Zn enhanced with the increasing methanol concentration. In the 4% v/v methanol solution, the signal intensity of Se was enhanced by a factor of 2.2, which is 1.3 for As and 1.1 for Zn, respectively. Further increase the concentration of the methanol further enhanced the signal intensity. However, the extent of this enhancement was less pronounced. This result could support our hypothesis, suggesting that C may enhance the ionization of hard-to-ionize elements in MICAP-MS. However, additional research is needed to elucidate the underlying principles of this effect, since methanol could not totally simulate the matrix in serum samples.

### Characterization of the serum samples with isotope dilution analysis

To investigate the performance of MICAP-MS for ID analysis, isotope ratios of ^40^Ca/^44^Ca, ^57^Fe/^56^Fe, and ^82^Se/^80^Se were measured to determine the Ca, Fe, and Se mass concentrations in the corresponding serum samples. Sample bracketing was performed with a solution containing naturally abundant Ca, Fe, and Se to correct the mass bias and signal drift. The average values of the triplicate ID analysis were compared with the reference values and those obtained with external calibration (see Table 2). The metrological compatibility of the data with the certified values was evaluated by calculating their *En* value. The results are considered metrologically compatible, if the absolute value of the *En* value is less or equal to 1 [18, 38].

**Table 2:** Comparison of Ca, Fe, and Se mass concentrations in serum samples determined by ID analysis and external calibration with the reference values. Unit for Ca and Fe, mg L^−1^; for Se, µg L^−1^

Elements		Reference		Isotope dilution			External calibration		
		Mean	*U*	Mean	*U*	*En*	Mean	*U*	*En*
BCR 304	Ca	88.04	0.76	90.07	1.57	0.6	89.02	2.02	0.4
BCR 637	Se	81	7	84	6	0.2	1232	84	14
BCR 638	Se	104	7	102	3	− 0.1	1467	90	15
BCR 639	Se	133	12	133	3	0	2279	138	15
NIST 909C	Ca	101	1.1	103	2.56	0.6	104	4	0.4
	Fe	0.903	0.039	0.938	0.045	0.4	0.941	0.08	0.2
	Se	118.7	3.3	120.5	3.2	0.3	1552	68	12
ClinChek 1	Fe	0.859	0.13	0.858	0.06	0	0.89	0.1	0.1
	Se	57.7	11.6	55.5	4	− 0.1	897	59	7
ClinChek 2	Fe	1.48	0.22	1.50	0.04	0.1	1.44	0.08	− 0.07
	Se	105	21	108	2.11	0.1	1172	72	13
Seronorm L1	Ca	97	20	103	1.9	0.2	106	5	0.2
	Fe	1.4	0.28	1.5	0.074	0.2	1.59	0.22	0.3
	Se	95	19	92	8	− 0.1	1296	101	11
Seronorm L2	Ca	138	28	133	2	− 0.1	136	6	− 0.04
	Fe	2.07	0.42	2.25	0.086	0.2	2.22	0.16	0.2
	Se	139	28	132	4	- 0.1	3560	162	20

Table 2 reveals that the Ca and Fe mass concentrations determined with ID analysis and external calibration were comparable and matched closely with the reference values. Compared to those obtained with external calibration, the Se mass concentrations determined with ID analysis align well with the reference values. This suggests that ID analysis was not obviously influenced by the serum matrix. All of the *En* values from the ID analysis were below 1, which confirms the metrological compatibility of these results

## Conclusions

We found that MICAP-MS combined with ID analysis proves to be a reliable technique for precise Ca, Fe, and Se quantification in blood serum. Different from Ar-based ICP-MS, MICAP-MS allows the determination of the most abundant isotopes of these elements, which generally interfere with Ar, thereby facilitating their use in ID analysis.

The matrix tolerance of MICAP-MS to Na was investigated by measuring the intensity recovery of ^6^Li, ^45^Sc, ^89^Y, ^115^In, ^159^ Tb, and ^209^Bi at increasing Na concentration. Like Ar-based ICP-MS, high Na concentration could result in intensity suppression in MICAP-MS, possibly due to the plasma loading and the space charge effect. At 50 mg L^−1^ Na concentration, the intensity recovery for most elements ranged between 70 and 75%. Over 60% suppression was found at a Na concentration of 2 g L^−1^. The Na matrix did not significantly affect the isotope ratios of ^57^Fe/^56^Fe and ^82^Se/^80^Se. However, higher Na concentration was found to cause isotopic fractionation for ^40^Ca and ^44^Ca, which might result from the space charge effect.

By external calibration, the obtained mass concentrations of most elements were consistent with their reference values. However, overestimation was observed in the results of Cr, Zn, As, and Se. The interferences of ^52^Cr could result from polyatomic species ^40^Ca^12^C^+^, ^40^Ar^12^C^+^, and ^35^Cl^16^O^1^H^+^, with ^40^Ca^12^C^+^ being the most likely contributor. The overestimation of Se, As, and Zn might be due to a signal enhancement effect, likely caused by the organic residues in the samples. Additional investigations performed with methanol showed a similar effect, supporting this assumption.

By ID analysis, the obtained Ca, Fe, and Se mass concentrations in the nine reference serum samples were comparable with the reference values. Most of the absolute values of *En* were below 1, confirming the metrological compatibility of the results. Combining MICAP-MS with ID analysis reduced the influences of matrix effects, enabling the analysis of samples in complex matrix effectively. Further research could build upon this work by delving into the carbon-containing matrix, potentially extending the applicability of MICAP-MS even further.

### Supplementary Information

Below is the link to the electronic supplementary material.

Supplementary file1 (DOCX 41 KB)

## Data Availability

Data available on request from the authors.

## Declarations

### Source of biological materials

Human serum certified reference materials were acquired from commercial providers.
