# Supplementary material for: Determination of calcium, iron, and selenium in human serum by isotope dilution analysis using nitrogen microwave inductively coupled atmospheric pressure plasma mass spectrometry (MICAP-MS)
Source: Anal Bioanal Chem. 2024 Apr 8;416(13):3117–25. doi: 10.1007/s00216-024-05274-0 (PMC11068692; doi:10.1007/s00216-024-05274-0)
Supplement: Supplementary file 1 — Supplementary file1 (DOCX 41 KB) [file 216_2024_5274_MOESM1_ESM.docx]

**Supporting Information**

Determination of calcium, iron, and selenium in human serum by isotope dilution analysis using nitrogen microwave inductively coupled atmospheric pressure plasma mass spectrometry (MICAP-MS)

Zengchao You*^1^, Alexander Winckelmann^1,2^, Jochen Vogl^1^, Sebastian Recknagel^1^, Carlos Abad*^1^

^1^Bundesanstalt für Materialforschung und -prüfung (BAM), Richard-Willstätter-Straße 11, D-12489 Berlin, Germany.

^2^Humboldt-Universität zu Berlin, Brook-Taylor-Straße 2, D-12489 Berlin, Germany.

E-Mail: Zengchao.You@bam.de and [Carlos.Abad@bam.de](mailto:Carlos.Abad@bam.de)

List of Tables

1. Comparison of the certified mass concentrations of the elements in serum samples (bold) with the values determined with external calibration. ………………………………………………………S1
2. Masses and mass concentrations of the Ca, Fe, and Se spike solutions used for each serum sample ………………………………………………………………………….…………….........S2
3. Masses and mass concentrations of the single-element solutions used for reverse ID ……………..S3
4. Corresponding IS and the calibration levels used for external calibration...………………………..S4
5. Densities of samples and spike solutions determined through weighing………………………….S5
6. LOD and LOQ for matrix-matched external calibration (in 50 mg L^-1^ NaCl).…………..………..S6

Table S1: Comparison of the certified mass concentrations of the elements in serum samples (bold) with the values determined with external calibration. Unit for Li, Mg, K, Ca, Fe, Cu, and Zn: mg L^-1^, Unit for Cr, Mn, As, and Se: µg L^-1^. n.d. for not determined. The uncertainty provided with each value is the expanded uncertainty, which was set to be twice the standard deviation.

|  | **Li** | **Mg** | **K** | **Ca** | **Fe** | **Cu** | **Zn** | **Cr** | **Mn** | **As** | **Se** |
| --- | --- | --- | --- | --- | --- | --- | --- | --- | --- | --- | --- |
| **BCR 304** | ***5.91 ±***  ***0.174*** | ***44.4 ±***  ***0.72*** | ***n.d.*** | ***88.04 ±***  ***0.75*** | ***n.d.*** | ***n.d.*** | ***n.d.*** | ***n.d.*** | ***n.d.*** | ***n.d.*** | ***n.d.*** |
|  | 6.83 ±  0.51 | 43.4 ±  2.4 | 226 ±  16 | 89.02 ±  2.02 | 1.5 ±  0.8 | 0.98 ± 0.18 | 10.6 ± 4 | 151 ±  16 | 16.4 ± 2.1 | 298 ± 18 | 5341 ± 190 |
| **BCR 637** | ***n.d.*** | ***n.d.*** | ***n.d.*** | ***n.d.*** | ***n.d.*** | ***n.d.*** | ***1.11 ±***  ***0.22*** | ***n.d.*** | ***n.d.*** | ***n.d.*** | ***81 ±***  ***7*** |
|  | 0.01 ±  0.02 | 15.7 ±  3.1 | 124 ±  14 | 49.4 ±  5.2 | 0.56 ± 0.32 | 0.9 ± 0.16 | 2.49 ±  0.62 | 155 ±  16 | 6.1 ± 1.4 | 90 ±  20 | 1232 ±  84 |
| **BCR 638** | ***n.d.*** | ***n.d.*** | ***n.d.*** | ***n.d.*** | ***n.d.*** | ***n.d.*** | ***1.43 ±***  ***0.21*** | ***n.d.*** | ***n.d.*** | ***n.d.*** | ***104 ±***  ***7*** |
|  | 0.06 ±  0.02 | 16 ±  2.4 | 120 ±  20 | 69 ±  5 | 1.1 ±  1 | 0.66 ±  0.14 | 2.45 ±  0.48 | 169 ±  18 | 8.4 ±  2 | 90 ±  12 | 1467 ±  90 |
| **BCR 639** | ***n.d.*** | ***n.d.*** | ***n.d.*** | ***n.d.*** | ***n.d.*** | ***n.d.*** | ***2.36 ±***  ***0.14*** | ***n.d.*** | ***n.d.*** | ***n.d.*** | ***133 ±***  ***12*** |
|  | 0.05 ±  0.02 | 15.6 ±  2.2 | 115 ±  20 | 56.2 ±  3.6 | 0.95 ± 0.12 | 0.85 ±  0.14 | 3.67 ±  0.31 | 88 ±  14 | 6.2 ±  1.4 | 110 ±  16 | 2279 ±  138 |
| **NIST 909C** | ***n.d.*** | ***21.76 ±***  ***0.16*** | ***162.7 ±***  ***2.7*** | ***101 ±***  ***1.1*** | ***0.903 ±***  ***0.039*** | ***n.d.*** | ***n.d.*** | ***n.d.*** | ***n.d.*** | ***n.d.*** | ***118.7 ±***  ***3.3*** |
|  | 0.04 ±  0.02 | 22.14 ±  2.2 | 167 ±  10 | 104.2 ±  4.2 | 0.941 ±  0.08 | 0.83 ± 0.15 | 2.64 ± 0.84 | 65 ±  12 | 7.2 ±  4 | 92 ±  11 | 1552 ±  68 |
| **ClinChek 1** | ***3.76 ± 0.57*** | ***16 ±***  ***1.6*** | ***n.d.*** | ***n.d.*** | ***0.859 ± 0.13*** | ***0.743 ± 0.111*** | ***1.22 ± 0.18*** | ***1.58 ± 0.4*** | ***2.41 ± 0.6*** | ***9.48 ± 1.9*** | ***57.7 ± 11*** |
|  | 3.24 ± 0.41 | 15.4 ±  1.82 | 102 ±  12 | 98 ±  13 | 0.89 ± 0.1 | 0.74 ± 0.11 | 2.61 ± 0.62 | 4.6 ± 1.4 | 1.99 ± 0.33 | 73 ±  16 | 897 ± 59 |
| **ClinChek 2** | ***7.58 ± 1.14*** | ***21.8 ± 2.2*** | ***n.d.*** | ***n.d.*** | ***1.48 ± 0.22*** | ***1.4 ± 0.21*** | ***1.7 ± 0.25*** | ***5.92 ± 1.19*** | ***6.24 ±***  ***1.25*** | ***19.3 ± 3.9*** | ***105 ± 21*** |
|  | 6.74 ± 0.81 | 19.8 ± 2.2 | 101 ±  10 | 79 ±  9 | 1.44 ± 0.08 | 1.28 ± 0.2 | 1.66 ± 0.75 | 12.7 ± 3.1 | 4.8 ±  0.84 | 70 ±  10 | 1172 ± 72 |
| **Seronorm L1** | ***5.06 ± 1.02*** | ***19.3 ±***  ***3.9*** | ***148 ± 30*** | ***97 ±***  ***20*** | ***1.4 ± 0.28*** | ***1.12± 0.23*** | ***1.46 ± 0.29*** | ***1.5 ±***  ***0.3*** | ***9.4 ±***  ***1.8*** | ***n.d.*** | ***95 ±***  ***19*** |
|  | 5.18 ± 0.62 | 18.4 ±  2.1 | 138 ±  12 | 106 ±  5.2 | 1.59 ± 0.22 | 1.08 ± 0.08 | 2.9 ± 0.6 | 5.3 ±  1.52 | 8.12 ±  1.4 | 170 ±  26 | 1296 ±  101 |
| **Seronorm L2** | ***10.0 ± 2.0*** | ***35.7 ±***  ***7.2*** | ***235 ±***  ***47*** | ***138 ±***  ***28*** | ***2.07 ± 0.42*** | ***2.18 ± 0.43*** | ***2.09 ± 0.42*** | ***3.91 ± 0.79*** | ***14.2 ± 2.9*** | ***n.d.*** | ***139 ± 28*** |
|  | 9.32 ± 0.84 | 33.4 ±  4.2 | 210 ±  16 | 136.2 ±  6.1 | 2.22 ± 0.16 | 2.17 ± 0.24 | 3.35 ± 0.7 | 10.6 ± 3.2 | 9 ±  3.2 | 142 ±  28 | 3560 ± 162 |

Table S2: Masses and mass concentrations of the Ca, Fe, and Se spike solutions used for each serum sample. Unit: g. n.d. for not determined.

|  | **Ca (529.25 mg L^-1^)** | **Fe (11.02 mg L^-1^)** | **Se (100.78 µg L^-1^)** |
| --- | --- | --- | --- |
| **BCR 304** | ***0.072*** | ***n.d.*** | ***n.d.*** |
| **BCR 637** | ***n.d.*** | ***n.d.*** | ***0.3234*** |
| **BCR 638** | ***n.d.*** | ***n.d.*** | ***0.4142*** |
| **BCR 639** | ***n.d.*** | ***n.d.*** | ***0.5285*** |
| **NIST 909C** | ***0.0818*** | ***0.0553*** | ***0.4605*** |
| **ClinChek 1** | ***n.d.*** | ***0.0547*** | ***0.4167*** |
| **ClinChek 2** | ***n.d.*** | ***0.0868*** | ***4161*** |
| **Seronorm L1** | ***0.0799*** | ***0.0877*** | ***0.3773*** |
| **Seronorm L2** | ***0.1224*** | ***0.1328*** | ***0.5561*** |

Table S3: Masses and mass concentrations of the single-element solutions used for reverse ID.

|  | **Mass** | **Mass concentration** |
| --- | --- | --- |
| **Ca** | ***0.0154 g*** | ***100.48 m*g L^-1^** |
| **Fe** | ***0.1053 g*** | ***10.23 m*g L^-1^** |
| **Se** | ***0.8110 g*** | ***100.90* µg L^-1^** |

Table S4: Corresponding IS and the calibration levels used for external calibration.

|  | **Measured m/z** | **Internal standard** | **Calibration levels (µg L^-1^)** |
| --- | --- | --- | --- |
| **Li** | ***7*** | ***^6^Li*** | ***0.1 - 10*** |
| **Mg** | ***24*** | ***^45^Sc*** | ***10 - 500*** |
| **K** | ***39*** | ***^45^Sc*** | ***10 - 500*** |
| **Ca** | ***40, 44*** | ***^45^Sc*** | ***10 - 500*** |
| **Fe** | ***56, 57*** | ***^45^Sc*** | ***0.1 - 10*** |
| **Cu** | ***63, 65*** | ***^45^Sc*** | ***0.1 - 10*** |
| **Zn** | ***64, 66*** | ***^89^Y*** | ***0.1 - 10*** |
| **Cr** | ***52, 53*** | ***^89^Y*** | ***0.1 - 10*** |
| **Mn** | ***55*** | ***^89^Y*** | ***0.1 - 10*** |
| **As** | ***75*** | ***^89^Y*** | ***0.1 - 10*** |
| **Se** | ***78, 80, 82*** | ***^89^Y*** | ***0.2 - 40*** |

Table S5: Densities of samples and spike solutions determined through weighing.

|  | **ρ (g mL^-1^)** |
| --- | --- |
| **BCR 304** | ***1.0100 ± 0.0050*** |
| **BCR 637** | ***1.00833 ± 0.00764*** |
| **BCR 638** | ***1.01333 ± 0.00578*** |
| **BCR 639** | ***1.01833 ± 0.00764*** |
| **NIST 909C** | ***1.02412 ± 0.00009*** |
| **ClinChek 1** | ***1.02167 ± 0.00577*** |
| **ClinChek 2** | ***1.02000 ± 0.00500*** |
| **Seronorm L1** | ***1.02333 ± 0.00289*** |
| **Seronorm L2** | ***1.02167 ± 0.00289*** |
| **Ca Spike** | ***1.00333 ± 0.00289*** |
| **Fe Spike** | ***1.00500 ± 0.00500*** |
| **Se Spike** | ***1.00167 ± 0.00289*** |

Table S6: LOD and LOQ for matrix-matched external calibration (in 50 mg L^-1^ NaCl).

|  | **m/z** | **LOD (µg L^-1^)** | **LOQ (µg L^-1^)** |
| --- | --- | --- | --- |
| **Li** | ***7*** | ***0.26*** | ***0.88*** |
| **Mg** | ***24*** | ***1.1*** | ***3.6*** |
| **K** | ***39*** | ***0.36*** | ***1.2*** |
| **Ca** | ***40*** | ***2.3*** | ***7.8*** |
| **Fe** | ***56*** | ***0.12*** | ***0.39*** |
| **Cu** | ***63*** | ***0.24*** | ***0.81*** |
| **Zn** | ***66*** | ***0.40*** | ***1.4*** |
| **Cr** | ***52*** | ***0.06*** | ***0.21*** |
| **Mn** | ***55*** | ***0.0016*** | ***0.0053*** |
| **As** | ***75*** | ***0.30*** | ***1.0*** |
| **Se** | ***80*** | ***0.70*** | ***2.4*** |
